# Environmental Uncertainty as a Moderator of Entrepreneurship Orientation and Innovation Capability during the Pandemic: A Case of Written Batik SMEs in Yogyakarta

**DOI:** 10.12688/f1000research.53433.1

**Published:** 2021-08-23

**Authors:** Humam Santosa Utomo, Susanta Susanta

**Affiliations:** 1 Business Administration, Universitas Pembangunan Nasional Veteran Yogyakarta, Sleman, Yogyakarta, Indonesia

**Keywords:** entrepreneurship orientation, innovation capability, environmental uncertainty, written batik

## Abstract

**Background: **Written batik is one of Indonesia's cultural heritages that must be preserved. During the Covid-19 pandemic, written batik producers in Indonesia experienced increasing challenges due to the uncertainty of the business environment. Innovation is a solution to the problems faced by SMEs during a pandemic to survive. This study aims to examine the effect of entrepreneurship orientation on innovation capability. This study also examines the role of moderating environmental uncertainty in strengthening the effect of entrepreneurship orientation on innovation capability.

**Methods:** The research was conducted at the centre of small and medium-sized batik companies Giriloyo, Bantul, Yogyakarta, Indonesia. Respondents of this study were 130 small and medium companies selected by random sampling. The questionnaire is used as a primary data collection tool. WarpPLS has been used to analyze the effect between variables in this study.

**Results:** Entrepreneurial orientation is an important aspect in improving the innovation capabilities of SMEs. Environmental uncertainty during the COVID-19 pandemic has strengthened the effect of entrepreneurial orientation on innovation capabilities.

**Conclusions:** The findings of this study indicate that entrepreneurial orientation has a significant effect on innovation capabilities. Environmental uncertainty significantly strengthens the effect of entrepreneurship orientation on innovation capability.

## Introduction

Written batik is one of the traditional products developed by SMEs (Small and medium-sized enterprises) in Yogyakarta, Indonesia. Indonesian batik was officially recognized by UNESCO on October 2, 2009 as an Intangible Cultural Heritage (ICH) at the UNESCO meeting in Abu Dhabi. Indonesian batik consists of written batik, printed batik, and combination batik. Written batik has a higher level of uniqueness than printed batik, so it requires a longer processing time. Giriloyo is an area where people make written batik their main livelihood. The Batik center in Giriloyo maintains and develops written batik.

During the Covid-19 pandemic, most SMEs experienced the problem of decreasing sales due to a reduced number of buyers. Limitations on social interaction and travel has caused a decrease in tourists visiting Yogyakarta so that the demand for batik products has decreased dramatically. Environmental uncertainty increases from time to time so it is a big challenge for SMEs. The strength of SMEs in sustaining business varies during a pandemic. Entrepreneurial orientation helps businesses survive by creating innovations that are relevant to a pandemic situation. Product innovation is needed during times of crisis to serve changing market demands. SMEs can also develop marketing innovations through online media.

Research examining the effect of entrepreneurial orientation on innovation has received much attention from previous researchers ^1– 3^. Environmental uncertainty has also been examined in regard to innovation and company performance ^4, 5^. However, studies on environmental uncertainty as a moderating variable that strengthens or weakens the effect of entrepreneurship orientation on innovation during the crisis are still minimal. A company's external environment is predicted to influence the use of company resources in creating innovation. Specifically, this study aims to examine the moderating effect of environmental uncertainty on the effect of entrepreneurial orientation on innovation capability. The formulation of the research problem is as follows:

RQ1: Does entrepreneurship orientation have an effect on innovation capability?

RQ2: Does environmental uncertainty moderate the effect of entrepreneurship orientation on innovation capability?

### Resource-based view theory

Resource-Based View (RBV) focuses on the concept of company attributes that are difficult to imitate as a source of superior performance and competitive advantage ^6, 7^. Thus, innovation is one of the keys to organizational success to develop and survive. The use of organizational resources is directed at achieving a sustainable competitive advantage to achieve the expected performance. According to Conner ^8^, firm performance variance depends on input ownership and the unique ability to utilize resources. The company's unique resources are difficult for competitors to imitate because they result from extended learning, a supportive cultural climate, and are not easy to buy. This study focuses on entrepreneurial orientation, which is one of the characteristics of a company as a company resource. A solid entrepreneurial orientation encourages the creation of innovations to increase company competitiveness.

### Entrepreneurship orientation

Entrepreneurship is the ability to think creatively and act innovatively as a basis, resource, and process to face lifestyle challenges ^9^. Zahra & Shaker define entrepreneurship as innovation and strategic renewal ^10^. Shane suggests that entrepreneurial processes stem from perceptions about the availability of opportunities or situations in which resources are transformed into profitable businesses ^11^. Entrepreneurial orientation is the view of an entrepreneur which is then implemented in managing a business through creative activities, the courage to take advantage of opportunities while taking risks, and not giving up on difficult situations. Therefore entrepreneurship orientation has a positive effect on the creation of innovative products and company performance.

### Environmental uncertainty

The rapidly changing business environment is a challenge for entrepreneurs. Changes in the business environment create uncertainty. Miller revealed that environmental uncertainty refers to the perception of the uncertainty of environmental variables that impact organizational performance ^12^. Changes in markets, technology, and the regulatory environment are all factors that cause environmental uncertainty ^13, 14^. Market uncertainty refers to market uncertainty, changes in market structure, and the level of competition within an industry ^13^. Regulatory uncertainty refers to the uncertainty of the actions of regulatory agencies that create and enforce regulations and policies ^14^. SMEs can have difficulty understanding the environment and this places SMEs in challenging situations related to strategic decision making ^15, 16^. The lack of information held by SMEs further hinders SMEs in making comprehensive decisions ^17^ which can lead to severe errors in decision making ^15, 16, 18^. SMEs continue to align strategies to change the changing environment that is currently full of uncertainty ^19^.

### Innovation capability

Innovation has been generally recognized as a critical factor for company success in terms of sales growth, profit, and competitiveness ^20, 21^. Innovation capability is conceptualized into two types, namely innovation as a process and innovation as a result ^22^. This study emphasizes the importance of innovation as a process. Hult *et al.* describe innovation as new processes, products, and ideas from the organization ^23^. Thornhill defines innovation as a process that begins with an idea, namely the development of findings and the introduction of new products, processes and new services in the market ^24^. Each company has different abilities in creating innovation. The ability to innovate describes how much strength the company has in creating creative ideas and innovations. The capability for innovation shows how much the company is able to develop new products according to market demand ^25^. Innovation capability in the context of this research is the ability of SMEs to develop new processes, new products, or new services according to market demand during the crisis due to the Covid-19 pandemic.

### Entrepreneurship orientation and innovation capability

Entrepreneurial orientation directs the behaviour of SMEs to create innovations. Entrepreneurship-minded SMEs dare to create new products needed by the market to improve market performance marked by opening new markets and increasing sales. Lee & Hsieh found that entrepreneurship has a significant influence on innovation and performance ^26^. The findings of Lin *et al.* on textile companies in Taiwan also show that entrepreneurial intensity affects innovation capabilities ^27^. Thus, the following hypothesis can be formulated:

H1: Entrepreneurship orientation has a significant effect on innovation capability.

### Environmental uncertainty as a moderator

High environmental uncertainty causes difficulties for SMEs in determining marketing strategies so that the innovations that are set may not be following rapidly changing market demands ^19^. The acceleration of SMEs in implementing innovation strategies is not proportional to the rapid changes in the market caused by uncertain regulations. This of course also has an impact on the marketing performance of SMEs. However, an unyielding attitude, the courage to take risks, and the courage to take advantage of every opportunity will actually trigger an entrepreneurial spirit to create new innovations during crisis times. Absorption power is stronger in external environments that are in high dynamism compared to environments with low dynamism ^2^. Thus, environmental uncertainty is predicted to strengthen the effect of entrepreneurship orientation on innovation capability during the pandemic. Thus, the following hypothesis can be formulated:

H2: Environmental uncertainty significantly strengthens the effect of orientation on innovation capability.

## Methods

This type of research is explanatory research, namely research conducted to investigate the relationship between variables. This study aims to investigate and explain the causal relationship between variables by testing hypotheses as well as providing explanations. The variables of this research consist of entrepreneurship orientation and innovation capability as an independent variable and dependent variables. The effect of these two variables is moderated by environmental uncertainties. A quantitative approach was used in analyzing this research ^28^. The entrepreneurship orientation variable refers to Sulistyo & Siyamtinah ^9^ and Shane ^11^. Variable innovation capability is based on Thornhill ^24^. Environmental uncertainty variables refer to Bstieler ^13^ and Engau *et al.* ^14^. The research was conducted in Bantul district, Yogyakarta Special Region province, Indonesia.

The population of this study were all batik SMEs in the Giriloyo batik centre, as many as 520 Batik SMEs. Written approval from the Chairperson of the Giriloyo Written Batik Group was obtained in accordance with document 8/VIII/2020. The research sample of 130 respondents was randomly selected using the Excel application. Sample size refers to the suggestion of Hair *et al.* ^29^ that the sample size is at least 100 respondents if the model contains five or fewer constructs. All respondents filled out the questionnaire completely. The data collection tool is a questionnaire with a Likert scale ranging from strongly disagree to strongly agree ^30^. Testing the validity and reliability of the instrument involved 30 respondents. The questionnaire was distributed in August 2020 offline by visiting respondents one by one to fill out the questionnaire and received approval from the Chairperson of the Batik Giriloyo SME group. Data processing used WarpPLS 6.0 ^31^ to test the effect between variables in the structural model. Equivalent functions can be caried out on the open-source software R ( R Project for Statistical Computing, RRID:SCR_001905).

## Results

### Validity and reliability of the instrument

Table 1 shows the coefficient of all items ≥ 0.3 so it can be stated that the instrument produces valid data (Sekaran, 2011). The Cronbach coefficient ≥ 0.6 indicates the instrument used is reliable (Malhotra, 2010).

**Table 1.** Result of instrument validity test.

Variable	Indicator	Correlation Coefficient	Cronbach’s Alpha
Entrepreneurship Orientation	Market orientation	0.684	0.765
	Think creatively	0.723	
	Effective use of company resources	0.580	
	Courage takes advantage of every opportunity	0.632	
	The courage to take risks	0.873	
	never give up on difficult situations	0.596	
Innovation Capability	Ability to create new products innovation	0.931	0.808
	Ability to create new services	0.905	
Environmental Uncertainty	Market changes	0.921	0.833
	Changes in the regulatory environment	0.931	

### Profile of Respondents

Table 2 shows that the majority of respondents were male (76.15%), most of the entrepreneurs are 26–45 years old (53.06%). Most of the companies were > 10 years old (45.39%) and most of the respondents were married (84.62%).

**Table 2.** Characteristics of the sample (% of respondents, n = 130).

Gender	Male	76.15
	Female	23.85
Entrepreneur age	18–25 years	16.15
	26–45 years	53.08
	>45 years	30.77
Firm age	3–6 years	19.23
	7–10 years	35.38
	> 10 years	45.39
Marital status	Single	15.38
	Married	84.62

### Final Structural Model

Measurement of model fit and quality indices refers to the WarpPLS analysis tool ^31, 32^. The measurement results show the following. Average Path Coefficient (APC) was 0.328, p < 0.001; average R-squared (ARS) was 0.258, p < 0.001; average adjusted R-square (AARS) was 0.248, p < 0.001; average block VIF (AVIF) was 1.022, acceptable if ≤ 5; average full collinearity VIF (AFVIF) was 1.901, acceptable if ≤ 5; Tenenhaus GoF (GoF) was 0.316, acceptable if ≥ 0.25; Sympson’s Paradox Ratio (SPR) was 1,000, acceptable if ≥ 0.7; Statistical Suppression Ratio (SSR) was 1,000, acceptable if ≥ 0.7; Nonlinear Bivariate Causality Direction Ratio (NLBCDR) was 1.000, acceptable if ≥ 0.7. These results indicate that the model is supported by good data and has quality indicators that meet the requirements in the WarpPLS.

### Hypothesis Testing

Hypothesis 1 states that entrepreneurship orientation influences innovation capability. The results ( Table 3) show that *p-*value is <0.000, so hypothesis 1 is accepted. Positive coefficients indicate that entrepreneurship orientation has a significant positive effect on innovation capability.

**Table 3.** Hypothesis testing results.

Relations between variables		Coefficient	*p*-value	Hypothesis Decision
Entrepreneurial Orientation	Innovation Capability	0.412	< 0.000	Accepted
Entrepreneurial Orientation * Environmental Uncertainty	Innovation Capability	0.245	< 0.000	Accepted

* significant at the 0.01 level

Hypothesis 2 states that environmental uncertainty significantly strengthens the effect of entrepreneurship orientation on innovation capability. The results ( Table 3) show that the p-value is <0.000, so hypothesis 2 is accepted. Positive coefficients indicate that environmental uncertainty strengthens the effect of entrepreneurship orientation on innovation capability.

## Discussion

**Figure 1.**
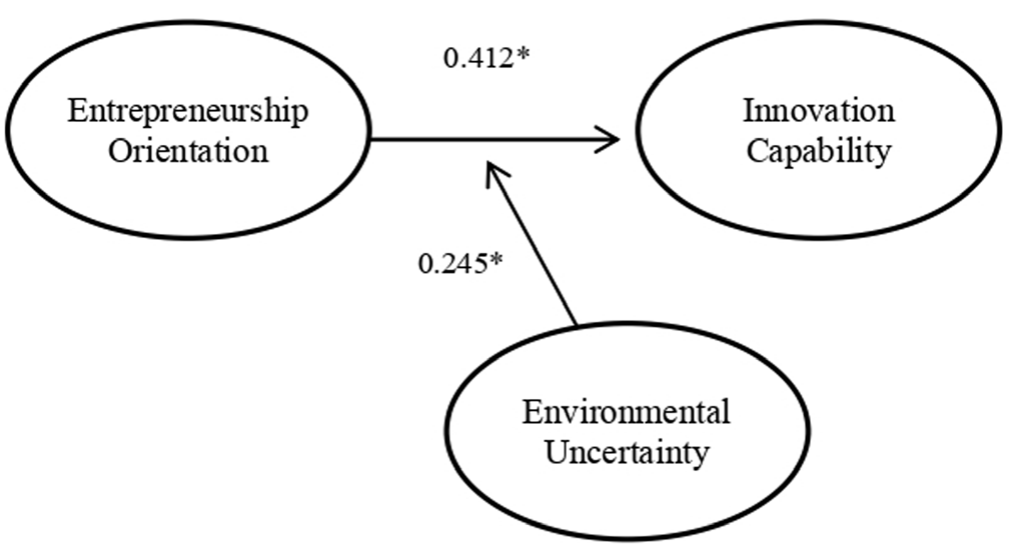
Final Structural Model.

The results of this study reveal two essential things in creating innovation and contributing to the field of entrepreneurship (see Figure 1). First, entrepreneurship orientation has a significant effect on innovation capability. The results of this study reinforce the argument of RBV ^6, 7^. According to RBV, competitive advantage and superior performance are the result of company attributes that are difficult to imitate by competing companies. One of the attributes or resources the company has is an entrepreneurship orientation. Entrepreneurship orientation enhances the company's innovation capabilities. These results are consistent with Lee & Hsieh who found that entrepreneurship directly affects innovation and performance ^26^. These results support the research of Lin *et al.* ^27^ on textile companies in Taiwan which concluded that high entrepreneurial intensity affects innovation ability and encourages sustainable innovation. Martínez *et al.* have also found that entrepreneurial orientation has a significant positive effect on company innovation ^1^. Entrepreneurial orientation possessed by entrepreneurs includes market orientation, creative thinking, utilizing company resources effectively, and the courage to take advantage of every opportunity, the courage to take risks, and never give up on difficult situations. Entrepreneurial orientation encourages proactive behaviour so that creativity and company innovation emerge.

Second, environmental uncertainty significantly strengthens the effect of entrepreneurial orientation on innovation capability. This finding shows that high environmental uncertainty is responded to positively by entrepreneurs who have a solid entrepreneurial orientation to create innovation. Uncertainty is considered a challenge that increases the entrepreneurial spirit to survive through product and service innovation. During the Covid-19 pandemic, written batik-producing SMEs in Yogyakarta experienced a very drastic decline in sales due to declining tourism. SMEs cannot sell their products at batik outlets so their turnover has dropped dramatically. Meanwhile, the government also makes regulations on social distancing so that direct contact between consumers and producers is minimized. Environmental uncertainty is a challenge for SMEs to protect their companies from bankruptcy. This environmental uncertainty does not reduce the entrepreneurial spirit, but instead increases the entrepreneurial spirit to create innovation.

In line with the findings of Zhai *et al.*, ^2^ the absorption capacity of SMEs is stronger in an external environment that has high dynamics compared to an environment with low dynamics. Efforts made by SMEs are creating product innovation and service innovation. SMEs not only add new product lines that consumers need during a pandemic, but also add online sales services through information and communication technology. SMEs adapt by utilizing online marketing techniques to serve consumers so that the marketing reach is broader and more flexible.

These findings provide a theoretical contribution. Environmental uncertainty does not always have a negative connotation, but instead triggers the ability to increase innovation. Environmental uncertainty strengthens the influence of entrepreneurship orientation on innovation ability. This finding is important and unique in the literature.

The results of this study have managerial implications for entrepreneurs and the government in fostering SMEs. Strengthening the entrepreneurial aspect must be emphasized on batik entrepreneurs because in an unstable condition, SMEs can still seize opportunities by creating innovations. Environmental uncertainty has actually spurred the spirit of SMEs to create new ideas in serving consumers. The government needs to increase its role in fostering SMEs, especially in conditions of uncertainty so that SMEs can immediately adapt to a rapidly changing environment.

## Conclusions

This study found that innovation capability was determined by entrepreneurship orientation. The views and attitudes of entrepreneurs encourage the creation of new innovations in the form of innovative products and new services. Environmental uncertainty actually strengthens the role of entrepreneurship orientation in creating innovation. Entrepreneurs learn and adapt to the rapidly changing business environment. These findings contribute to the management of SMEs during times of crisis. Based on a business orientation, environmental uncertainty actually increases innovation capabilities.

This study has limitations. First, this study was conducted during the Covid-19 pandemic and did not compare to regular times, meaning longitudinal studies are needed. Second, this study does not classify the level of environmental uncertainty so that the relationship between uncertainty level and innovation is unknown. Third, this paper discusses innovation and does not yet discuss SME performance. Various innovations carried out by SMEs have not certainly had positive implications for the performance of SMEs. Likewise, entrepreneurship orientation and environmental uncertainty may have different effects on SME performance. Therefore, further research is needed to obtain more comprehensive research results.

## Data availability

### Underlying Data

Figshare: a dataset of research results in written batik Giriloyo, Indonesia. https://doi.org/10.6084/m9.figshare.14563521.v1 ^32^

This project contains the following underlying data.

•   Data130 resp.cvc (questionnaire results)

•   KUESIONER.pdf (Blank research questionnaire).

•   RESULTS OF DATA PROCESSING.pdf (results of statistical analysis)

Data are available under the terms of the Creative Commons Zero "No rights reserved" data waiver (CC0 1.0 Public domain dedication).

## Ethical Consideration

This study was approved by the ethics commission for the human respondent at the Research and Community Service Institutions (LPPM) Universitas Pembangunan Nasional Veteran Yogyakarta (number: Sket/23/UN62.21/KL 00/VII/2020).

## Participant consent

Written informed consent for participation and for publication of their data was obtained from all participants. Written approval from the Chairperson of the Giriloyo Written Batik Group was obtained in accordance with document 8/VIII/2020. 
