# Supplementary material for: Environmental Uncertainty as a Moderator of Entrepreneurship Orientation and Innovation Capability during the Pandemic: A Case of Written Batik SMEs in Yogyakarta
Source: F1000Res. 2022 Nov 15;10:844. Originally published 2021 Aug 23. [Version 2] doi: 10.12688/f1000research.53433.2 (PMC9692048; doi:10.12688/f1000research.53433.2)
Supplement: Peer review history

# Peer review: Reviewer response for version 2

**DOI:** 10.5256/f1000research.140204.r155802

**Published:** 2022-11-24

**Authors:** Reni Shinta Dewi

**Affiliations:** 1 Universitas Diponegoro, Semarang, Indonesia

The revision is in accordance with the comments given.

Is the work clearly and accurately presented and does it cite the current literature?

Partly

If applicable, is the statistical analysis and its interpretation appropriate?

Partly

Are all the source data underlying the results available to ensure full reproducibility?

Yes

Is the study design appropriate and is the work technically sound?

Yes

Are the conclusions drawn adequately supported by the results?

Partly

Are sufficient details of methods and analysis provided to allow replication by others?

Partly

Reviewer Expertise:

Knowledge Management, RBV, HRM, Strategic Management

I confirm that I have read this submission and believe that I have an appropriate level of expertise to confirm that it is of an acceptable scientific standard.

# Peer review: Reviewer response for version 1

**DOI:** 10.5256/f1000research.56817.r150655

**Published:** 2022-09-21

**Authors:** Andreas Walmsley

**Affiliations:** 1 Plymouth Marjon University, Plymouth, UK

A timely study that adds to the literature seeking to understand and explain the relationship between environmental change and levels of innovation. The relationship between Entrepreneurial Orientation and Innovation Capability is explored and then Environmental Uncertainty is added as a moderator. The authors recognise some limitations of the study in the conclusion and yet there is sufficient originality and novelty here to merit publication. I think other researchers will be able to draw on these findings in testing some of the hypothesised relationship and also developing more complex models. That said, it is in part the simplicity of the model that is being tested that is one of the paper’s strengths.

 I have only one recommendation and that is that the authors are consistent in the use of either the term ‘entrepreneurial orientation’ or ‘entrepreneurship orientation’. Similarly, the author changes between innovation capability and innovation ability. I would recommend being consistent here too.

Is the work clearly and accurately presented and does it cite the current literature?

Yes

If applicable, is the statistical analysis and its interpretation appropriate?

Yes

Are all the source data underlying the results available to ensure full reproducibility?

Yes

Is the study design appropriate and is the work technically sound?

Yes

Are the conclusions drawn adequately supported by the results?

Yes

Are sufficient details of methods and analysis provided to allow replication by others?

Yes

Reviewer Expertise:

Entrepreneurship, Entrepreneurship Education, SMEs, Career Development

I confirm that I have read this submission and believe that I have an appropriate level of expertise to confirm that it is of an acceptable scientific standard.

# Peer review: Reviewer response for version 1

**DOI:** 10.5256/f1000research.56817.r92922

**Published:** 2021-09-07

**Authors:** Reni Shinta Dewi

**Affiliations:** 1 Universitas Diponegoro, Semarang, Indonesia

**Background:**

- All industries are affected by The Covid-19 Pandemic, but why only written batik is reviewed. Give the evidence support. The urgency of this research has not been explored. 

**Conceptual Model:**

- The model is not clearly especially  the explanation for the path of Environmental Uncertainty as a moderator variable.

**Method of Research:**

- The population is 520, but your sample is 130. Can you clarify how you got it. And How do you make sure your sample complies with random requirement. You should be defines the entrepreneurial orientation with clearly, because one of your dimension is market orientation. You should explain how to building the capability that direct connect with market orientation. 

**Discussion and Conclusion:**

- In your article said that: According to RBV, competitive advantage and superior performance are the result of company attributes that are difficult for competing companies to imitate. One of the attributes or resources owned by the company is an entrepreneurial orientation.
- Prove the theoretical and empirical support of the statement.
- What your advise for the future research? 
- What the managerial implication for the SME?

Is the work clearly and accurately presented and does it cite the current literature?

Partly

If applicable, is the statistical analysis and its interpretation appropriate?

Partly

Are all the source data underlying the results available to ensure full reproducibility?

Yes

Is the study design appropriate and is the work technically sound?

Yes

Are the conclusions drawn adequately supported by the results?

Partly

Are sufficient details of methods and analysis provided to allow replication by others?

Partly

Reviewer Expertise:

Knowledge Management, RBV, HRM, Strategic Management

I confirm that I have read this submission and believe that I have an appropriate level of expertise to confirm that it is of an acceptable scientific standard, however I have significant reservations, as outlined above.
